# Ceramide function in the brain: when a slight tilt is enough

**DOI:** 10.1007/s00018-012-1038-x

**Published:** 2012-06-24

**Authors:** Chiara Mencarelli, Pilar Martinez–Martinez

**Affiliations:** Department of Neuroscience, School for Mental Health and Neuroscience, Maastricht University, PO Box 616, 6200 MD Maastricht, The Netherlands

**Keywords:** Ceramide, Sphingolipids, Rafts, Apoptosis, Neurodegeneration

## Abstract

Ceramide, the precursor of all complex sphingolipids, is a potent signaling molecule that mediates key events of cellular pathophysiology. In the nervous system, the sphingolipid metabolism has an important impact. Neurons are polarized cells and their normal functions, such as neuronal connectivity and synaptic transmission, rely on selective trafficking of molecules across plasma membrane. Sphingolipids are abundant on neural cellular membranes and represent potent regulators of brain homeostasis. Ceramide intracellular levels are fine-tuned and alteration of the sphingolipid–ceramide profile contributes to the development of age-related, neurological and neuroinflammatory diseases. The purpose of this review is to guide the reader towards a better understanding of the sphingolipid–ceramide pathway system. First, ceramide biology is presented including structure, physical properties and metabolism. Second, we describe the function of ceramide as a lipid second messenger in cell physiology. Finally, we highlight the relevance of sphingolipids and ceramide in the progression of different neurodegenerative diseases.

## Introduction

Ceramides are a family of lipid molecules that consist of sphingoid long-chain base linked to an acyl chain via an amide bond. Ceramides differ from each other by length, hydroxylation, and saturation of both the sphingoid base and fatty acid moieties.

**Fig. 1:**
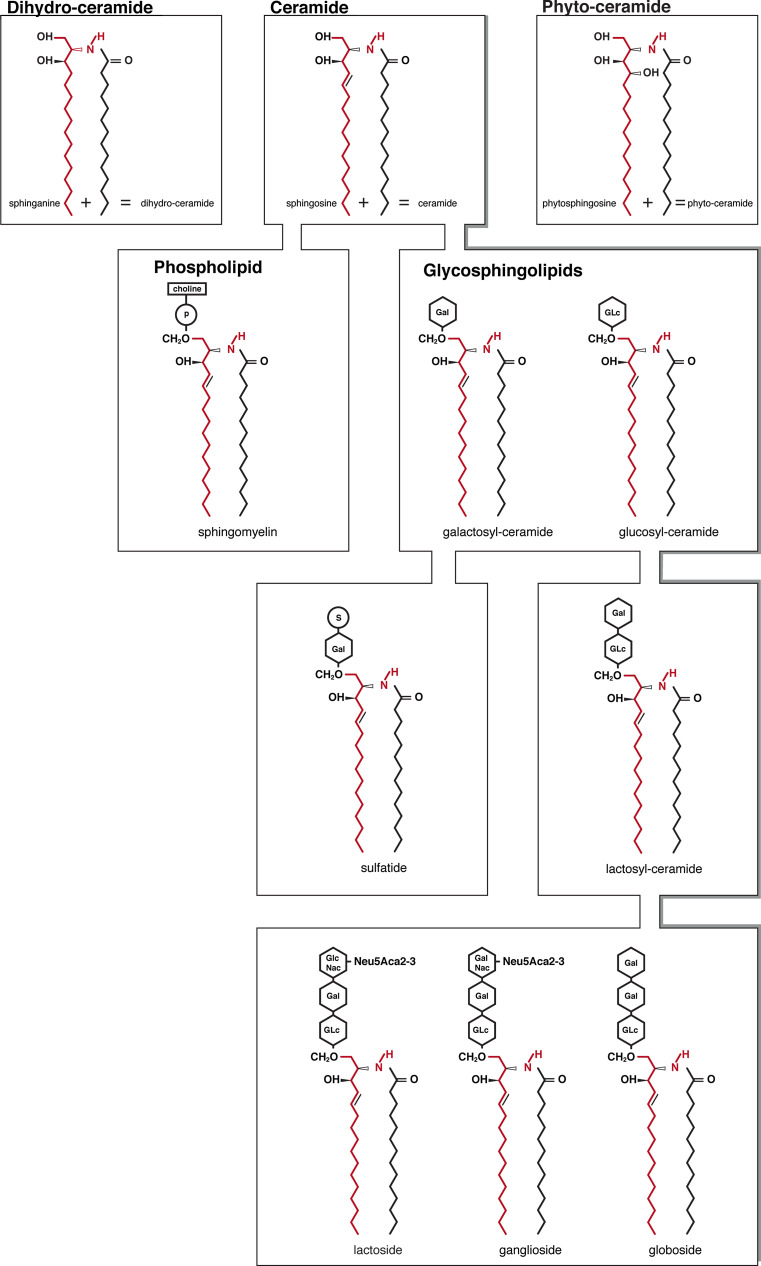
Chemical structure of sphingoid bases (sphinganine, sphingosine, phytosphingosine), ceramide species (dihydroceramide, ceramide and phytoceramide) and complex sphingolipids. Sphingomyelin, synthesized by the transfer of the phosphorylcholine moiety to the C-1 hydroxyl group of ceramides, is the only cell membrane phospholipid not derived from glycerol. Alternatively, modification of a ceramide by addition of one or more sugars directly connected at the primary alcohol group yields complex glycosphingolipids. Galactosylceramide and glucosylceramide (cerebrosides) have a single monosaccharide (galactose or glucose) as polar head group; sulfatides are the sulfuric acid esters of galactocerebrosides. Addition of a galactose to glucosylceramide gives rise to lactosylceramide, precursor of globo-, ganglio- and lactosides. Globosides contain multiple sugar moieties. Ganglio- and lactosides have a complex oligosaccharide core structures with one or more sialic acids in the polar head

Sphingoid bases are of three general chemical types: sphingosine, dihydrosphingosine (commonly known as “sphinganine”, as it will be addressed in this review) and phytosphingosine. Based on the nature of the sphingoid base backbone, we can distinguish three main subgroups in the ceramide family: the compound named ceramide contains sphingosine, which has a* trans*-double bond at the C4–5 position in the sphingoid base backbone; dihydroceramide, the inactive precursor of ceramide, contains sphinganine, which presents a saturated sphingoid backbone devoid of the 4,5-*trans*-double bond; phytoceramide, the yeast counterpart of the mammalian ceramide, contains phytosphingosine, which has a hydroxyl group at the C4 position [1] (Fig. 1).

The fatty acid components of ceramides vary widely in composition, but they are typically long. Their acyl chain lengths range from 14 to 26 carbon atoms (or greater), although the most common fatty acids are palmitic (C16:0) and stearic (C18:0) non-hydroxy fatty acids. The fatty acids are commonly saturated or mono-unsaturated. α-Hydroxylated fatty acids (a hydroxyl group at the C-2 position) and ω-hydroxy fatty acid (a hydroxyl group on the terminal C atom) are often present as well [2].

### Activation of ceramide

Small changes in the molecular structure of ceramide moiety can regulate its biological function. Dihydroceramide is an early intermediate in the de novo ceramide biosynthesis. Considered the innocuous precursor of ceramide, dihydroceramide differs from ceramide only by reduction of the C4–5* trans*-double bond in the sphingoid backbone inhibiting [3] or reducing its biological activity [4] when compared with ceramide moiety. The introduction of a* trans*-double bond between C4 and C5 results in the bioactive molecule of ceramide. This reaction is catalyzed by the enzyme (dihydro)-ceramide desaturase, which is localized in the cytosolic leaflet of the endoplasmic reticulum (ER) membrane [5, 6]. In this way, cells can fine-tune the amount of biologically active ceramide. The presence of the double bond in the sphingosine chain determines the tilt of ceramides in the membrane and enables the lipid to interact with enzymes such as hydrolases and phosphatases [7].

Moreover, unsaturation in the sphingoid backbone augments intramolecular hydration/hydrogen bonding in the polar region. This may allow the close packing of the ceramide molecules, which exhibit a tighter intramolecular interaction than comparable lipids [8–10]. This higher packing density of ceramides within the lipid bilayer affects the physical properties of membranes [11].

### Short-chain ceramide

Synthetic short-chain ceramides (*N*-acyl chains of 2 to 8 carbon atoms) are commonly used to mimic the mechanisms of action of naturally occurring long-chain ceramides, which are highly hydrophobic compounds. Short-chain ceramides are water soluble and membrane-permeable and can be easily used as experimental tools within living cells [12–16]. Small amounts of C2-ceramide are normal components in brain (10 pmol/g) and liver (25 pmol/g) [17] although the metabolic origin and physiological activity of this short ceramide are uncertain.

NMR characterization of C-2 and C-18 ceramides showed that the conformation of the polar region of the two molecules is the same [9]. Since the interaction between ceramides and their ligand molecules probably occurs through the polar head, the maintenance of the headgroup conformation irrespective of *N*-acyl chain length is enough for C-2 ceramides to reproduce most of the long-chain ceramides signaling effects. However, the length of the fatty acyl chain modifies significantly the biophysical properties of the ceramide moieties [18] and in some reports long- and short-chain ceramides have been found to have different biological effects [19, 20].

The major difference between short and long ceramides is in the geometrical shapes they adopt at the membrane level that consequentially gives rise to different behaviors. The hydrophobic portion of C-2 is smaller than the polar headgroup. Therefore, C-2 has a shape that favors a positive curvature in lipid monolayer [21]. Long-chain ceramides are cone shaped molecules with opposite geometrical properties, which induce a negative curvature of the two halves of the bilayer towards the aqueous milieu, leading to membrane trafficking via vesiculation and fusion [22, 23]. Moreover, long-chain ceramides increase the order of the acyl chains in the bilayers, thus decreasing fluidity and stabilizing the membrane [24–26]. Conversely, short-chain ceramides perturb the structural order of the lipid bilayer. Long-chain ceramides are immiscible with phospholipids, while short-chain ceramides mix much better and are therefore able to spontaneously overcome membrane barriers [27]. Once inside the cell since they possess the appropriate stereochemistry, short ceramides might bind target proteins normally inaccessible for the longer species. On the contrary, naturally occurring long-ceramides are eminently hydrophobic even compared to other lipid species and as a consequence their concentrations in the cytosol are extremely low. This hydrophobicity of ceramides justifies the need for a ceramide transfer protein (CERT) in cells [28]. CERT localizes inside the cell and modulation of its activity may result in significant changes in ceramide levels [62]. Therefore, since short-chain ceramides behave as soluble amphiphiles [29], they are suspected to have cellular effects that cannot be extrapolated to natural ceramide species (mainly insoluble amphiphiles) and their use might lead to confusion on the role of ceramide in cellular signaling.

### Ceramides as precursors of sphingolipids

Free ceramides are molecules known to exert a wide range of biological functions in many of the most critical cellular events, including growth, differentiation, apoptosis and oncogenesis. Ceramides are the core structure of a class of complex lipid called sphingolipids, ubiquitous components of eukaryotic cell membranes [30]. Sphingolipids were initially described in brain tissue in the second half of the 19th century [31]. The name sphingolipids denotes their enigmatic (namely sphinx-like) nature that, despite intense research, still remains unclear. Sphingolipids have long been regarded as inactive and stable structural components of the membrane; however they are now well recognized to be biologically active in processes of cellular biology.

Sphingolipids are very heterogeneous and are classified depending on their structural combinations in long-chain (sphingoid) bases, amide-linked fatty acids [32] and hundreds of headgroup variants [33].

Sphingolipids are generated by attachment of different polar headgroups at the primary alcohol group (C1–OH) of a ceramide molecule. Depending on the type of polar group, two major classes are defined: phosphosphingolipids and glycosphingolipids (GSLs) (Fig. 1). The typical phosphosphingolipid in mammalian cells is sphingomyelin (SM), synthesized by the transfer of the phosphorylcholine moiety (from phosphatidylcholine) to the C1–OH of ceramides.

Alternatively, modification of a ceramide by addition of one or more sugars yields complex GSLs. As a result of the great heterogeneity in the glycan moiety, among GSLs much variation exists. When a single monosaccharide is present, the GSL is referred to as a cerebroside (also known as monoglycosylceramides). Usually glucose or galactoses are attached directly to the ceramide portion of the molecule, resulting in glucosylceramide (GlcCer; glucocerebroside) and galactosylceramide (galactocerebroside), respectively. The sulfuric acid esters of galactosylceramide are the sulfatides. Galactosylceramide and sulfatide are highly enriched in oligodendrocytes and myelin-forming cells compared to other membranes [34]. By contrast GlcCer is not normally found in neuronal cell membranes. Additionally, a galactose can be transferred by the enzyme lactosylceramide synthase to GlcCer to form lactosylceramide (LacCer) [35, 36], which plays a pivotal role as a precursor for the synthesis of complex GSLs [37]. In fact, the common LacCer structure is then elongated by different glycosyltransferases, thereby defining the classes of GSLs that are identified as ganglio-, globo-, lacto- and (neo)-lacto-subtypes according to their specific saccharide core structures.

Globosides represent cerebrosides that contain additional carbohydrates predominantly galactose, glucose or *N*-acetylgalactosamine (GalNAc). Gangliosides are very similar to globosides except that they also contain one or more sialic acid residues on their carbohydrate chains. Gangliosides comprise approximately 5 % of brain lipids and are mainly present in astroglia, followed by neurons and oligodendrocytes. Lacto and (neo)-lacto-series are GSLs classified on the basis of the core oligosaccharide structures present in their molecules and catalyzed by the transfer of *N*-acetylglucosamine (GlcNAc) onto LacCer [35]. Polar carbohydrate chains of GSLs extend toward the extracellular milieu, forming specific patterns on the surface of cells, contributing to cell recognition during differentiation, development and immune reaction [38]. These different types of sphingolipids can be converted back to ceramide by the removal of the polar headgroup by specific enzymes.

## Ceramide generation

Ceramides can be produced in cells either via the de novo synthesis or via hydrolysis of complex sphingolipids [39]. The activation of different catabolic enzymes yields ceramide within a few minutes whereas the de novo synthesis produces ceramide in several hours [40]. Different extra- and intra-cellular stimuli dictate the pathway used for ceramide generation resulting in distinct subcellular localization of ceramide and different biochemical and cellular responses.

### De novo synthesis of ceramide takes place in the ER

In animal cells, ceramide is de novo-synthesized on the cytoplasmic face of the smooth endoplasmic reticulum (ER) [5, 41] and in mitochondria [42, 43].

**Fig. 2:**
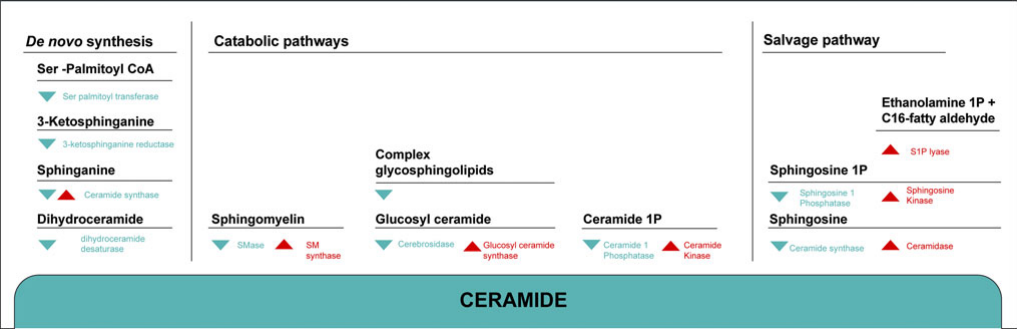
Overview of the metabolic pathways involved in the synthesis of endogenous ceramide. Ceramide can be formed by de novo synthesis, by degradation of complex SLs or by re-acylation of sphingoid long-chain bases (salvage pathway). The de novo pathway involves several enzymatic steps. Through catabolic pathways ceramide is generated by either hydrolysis of the membrane lipid SM by the SMase enzymes or by lysosomal breakdown of complex GSLs. Ceramide itself is degraded by ceramidase to regenerate sphingoid bases. The sphingosine formed is then phosphorylated and finally degraded to phosphoethanolamine and C16-fatty aldehyde by the action of S1P lyase. A salvage pathway uses the enzyme ceramide synthase to produce ceramide from sphingosine. Once generated, ceramide can serve as a substrate for the synthesis of SM and GSLs or be converted into various metabolites such as sphingosine or Cer1P

The de novo synthesis of ceramides in eukaryotes begins with the condensation of serine and palmitoyl-CoA to form 3-ketosphinganine, through the action of serine palmitoyl transferase (SPT) (Fig. 2). This enzyme is composed of two subunits: Lcb1 and Lcb2. Mutations in the human Lcb1 gene underlie hereditary autonomous neuropathy, a neurodegenerative disorder of the peripheral nervous system [44].

Subsequently, 3-keto-sphinganine is reduced to the sphingoid base sphinganine, which is subsequently* N*-acylated by (dihydro)-ceramide synthase (CerS) to form dihydroceramide. The enzyme (dihydro)-ceramide desaturase introduces the double bond to the position C4 to form mammalian type ceramides [6, 45].

CerS represents a key enzyme in the pathway for de novo sphingolipid biosynthesis. Interestingly, these highly conserved transmembrane proteins are also known as human homologues of yeast longevity assurance gene (LASS1).

Six different CerSs (CerS1–6) have been identified in vertebrates and plants [46], whereas most of the other enzymes involved in sphingolipids metabolism exist in only one or two isoforms [46]. Each CerS regulates the de novo synthesis of endogenous ceramides with a high degree of fatty acid specificity. In line with the presence of multiple CerSs, ceramides occur with a broad fatty acids length distribution inside the cell. Although some CerSs are ubiquitously expressed, other isoforms present a very specific distribution among tissues, according to the need of each tissue for specific ceramide species [47, 48]. CerS1 specifically generates C18 ceramide and is highly expressed in the brain and skeletal muscles but is almost undetectable in other tissues. CerS2 mainly generates C20–26 ceramides and has been found to have the highest expression of all CerSs in oligodendrocytes and Schwann cells especially during myelination. The selectivity of different CerS isoforms to synthesize different ceramide species is important since ceramides with specific acyl chain lengths might mediate different responses within cells [46]. Fumonisins are toxic mycotoxins with a very similar structure to sphingosine or sphinganine, which is a substrate for CerS. Since these fungal metabolites are able to inhibit CerS reaction, they are extensively used to study the role of ceramide generated through the de novo pathway in the ER [49]. On the contrary, the mitochondrial CerS is not affected by fumonisins, suggesting that its activity is distinct from the ER resident enzyme [42, 43].

Neo-synthesized ceramides subsequently traffic from the luminal face of the ER to the Golgi compartment where different polar heads are incorporated into the ceramide molecule to form complex sphingolipids [50].

### Ceramide transport from ER to the Golgi

The high hydrophobicity and low polarity of ceramide moiety limit free ceramide to circulate inside the cell or more generally in solution. This may explain the occurrence of several isoenzymes of ceramide biosynthesis at different subcellular sites and supports the view that the site of ceramide formation might determine its function.

On the other hand, the cell needs to transport ceramide from the ER to the Golgi compartment for the synthesis of GSLs and SM. Ceramides destined for conversion to GSLs appear to reach the Golgi only via the classical vesicular route [28]. The step-wise addition of sugar groups to ceramides is catalyzed by membrane bound glycosyltransferases and it is restricted to the ER-Golgi complex [51]. The synthesis of most GSLs begins with glucosylation of ceramide to form GlcCer, at the cytosolic surface of the Golgi [52]. The direction in which GlcCer is trafficked is controversial. GlcCer normally localizes to* trans*-Golgi and* trans*-Golgi network, whereas it remains in the* cis*-Golgi on the knockdown of FAPP2. Two inhibitors of intra-Golgi membrane trafficking did not affect the synthesis of GSLs. These observations suggest that GlcCer is transported from the* cis*-side of Golgi to the trans side by FAPP2 in a nonvesicular manner [53]. On the other hand, it has been suggested that GlcCer synthesized at the Golgi is retrogradely transported to the ER, where it is translocated to the lumen, and then transported to the Golgi again [54] for the subsequent synthesis of LacCer and more complex GSLs [55].

Ceramides destined for the formation of SM reach the Golgi carried by CERT in a non-vesicular manner [28, 56–58].

CERT mediates the transfer of ceramides containing C14–C20 fatty acids but not longer-chain ceramides [59]. This correlates with the presence of a C14–20 acyl chain SM in many tissues and cell lines whereas GSLs are formed by longer ceramides. CERT, works as mediator of sphingolipids homeostasis. Loss of functional CERT in Drosophila affects plasma membrane fluidity and increases oxidative stress [60] and CERT is critical for mitochondrial and ER integrity [61]. Interestingly, CERT has an alternatively spliced isoform characterized by the presence of an additional 26 amino acids domain, responsible for its localization at the plasma membrane and consequent secretion to the extracellular milieu, named CERT_L_ or Goodpasture antigen binding protein (GPBP) [62]. These two isoforms are differentially expressed during development. CERT_L_ is more abundant at early stages of embryonic maturation and its knockdown leads to severe developmental deficit in muscle and brain because of increased apoptosis [63]. As development progresses, the initially very low levels of CERT, gradually increase. Both isoforms can be detected in adult brain [64].

Other reports showed elevated CERT_L_ expression levels to be associated with several autoimmune disorder e.g., lupus erythematosus, multiple sclerosis, myasthenia gravis, Addison disease [65]. An efficient execution of apoptotic signaling is important to inhibit inflammation and autoimmune responses against intracellular antigens [66] and modulation of CERT/CERT_L_ levels has a direct influence in ceramide levels and could be responsible for balancing cell death during embryogenesis and under pathophysiological condition.

Once delivered to the Golgi apparatus, ceramide spontaneously translocates from the cytosolic to the luminal leaflet for SM synthesis. Formation of SM from ceramide is catalyzed by sphingomyelin synthase (SMS) [67] that transfers the phosphocholine headgroup from phosphatidylcholine onto ceramide yielding SM as a final product and diacylglycerol (DAG) as a side product [68]. If ceramide is a key metabolic intermediate for sphingolipids with an amide backbone, DAG is the precursor for glycerol-derived phospholipids and, as well as ceramide, it plays important roles in many signaling pathways. Whether the DAG generated by SMS regulates cellular processes remains unclear. SMS exists in two isoforms, SMS1, faces the lumen of the* cis*/medial Golgi [69, 70] and it is responsible for the de novo synthesis of SM [70]; SMS2, which resides in the plasma membrane [68, 71], could instead play a more specific role in signal transduction events. In neural cells the de novo SM is mostly synthesized at the plasma membrane and the production at the* cis* medial Golgi is less prominent [72, 73]. This indicates that the subcellular localization of SM formation is cell type specific and that SMS activities may be involved in different biological processes.

### Catabolic pathways for ceramide production

Beside the de novo pathway, significant contribution to intracellular ceramide levels occur also through hydrolysis of complex sphingolipids by activation of different hydrolases [74] (Fig. 2).

Ceramides derived from SM catabolism require the activation of sphingomyelinases (SMase) [75], specific forms of phospholipase C, which hydrolyze the phosphodiester bond of SM yielding water soluble phosphorylcholine and ceramide [76]. Several SMases have been characterized and classified by their pH optimum, subcellular distribution and regulation. The best-studied of these SMases is the acid sphingomyelinase (aSMase), which exhibits an optimal enzymatic activity at pH 4.5–5 [77]. This lipase is localized in lysosomes and is required for the turnover of cellular membranes [78]. ASMase is deficient in patients with the neurovisceral form (type A) of Niemann–Pick disease, with consequent abnormal accumulation of SM in many tissues of the body [79]. Besides this lysosomal/endosomal aSMase, a secreted zinc-activated form of aSMase was first identified in serum [80] and found to be secreted by many cell types [81, 82]. These two aSMases are differentially glycosylated and processed at the NH_2_-terminal (72) but they are products of the same gene [81]. Neutral SMases (nSMase) are membrane bound enzymes with an optimal activity at a neutral pH. Several isoforms have been characterized. NSMase 1 is localized in the membranes of the ER, [83, 84] and it is ubiquitously expressed and highly enriched in kidney [85]. NSMase 2 has a different domain structure than nSMase 1 and is specifically highly expressed in brain [86, 87] [88]. A third nSMase (nSMase 3) is ubiquitously present in all cell types and distributed mainly in the ER and Golgi membrane [89]. NSMases are further classified as Mg^2+^/Mn^2+^ dependent or independent. An alkaline SMase exists only in intestinal cells and it is activated by bile salts [90]. The function of these multiple isoforms is still elusive; however their membrane localization has lead to speculation that they may contribute to the modification of local microdomains in the membrane organization during vesicle formation, transport, and fusion [91, 92].

### Salvage pathway

Ceramides can be generated by an alternative acyl-CoA-dependent route (Fig. 2). This pathway relies upon the reverse activity of the enzyme ceramidase (CDase), which is called the “salvage pathway” since catabolic fragments are recycled for biosynthetic purposes [93, 94]. As the name suggests, CDase catalyses the hydrolysis of ceramide to generate free sphingosine and fatty acid. Together with ceramide production, CDase regulates also sphingosine levels. In fact, it is important to note that whereas sphinganine is generated by de novo sphingolipid biosynthesis (Fig. 2), free sphingosine seems to be derived only via turnover of complex sphingolipids, more specifically by hydrolysis of ceramide [5]. The catabolism of ceramide takes place in lysosomes from where sphingosine can be released [95] in contrast to ceramide, which does not appear to leave the lysosome [96]. Free sphingosine is probably trapped at the ER-associated membranes where it undergoes re-acylation (condensation with a fatty-acylCoA) to again generate ceramide. This “reverse” activity is carried out by the same CDase [96, 97].

As with SMase, different CDases have been identified associated with different cellular compartments according to the pH at which they achieve optimal activity (acid, neutral and alkaline). Acid CDases (aCDase) are lysosomal [98–100], whereas neutral/alkaline CDases (nCDase and alCDase) have been purified from mitochondria [42, 101] and nuclear membranes [102]. CDases have been isolated from soluble fractions of rat brain [103], mouse liver and human kidney. A purely alkaline CDase has been localized to the Golgi apparatus and ER [104, 105]. This variability in CDases subcellular localizations and distribution in tissues suggests that these enzymes may have diverse functions in the biology of the cell.

N/a CDases have been shown to catalyze the reverse reaction to generate ceramide from sphingosine and fatty acids [97, 104, 106, 107] whereas the acid isoform resides in lysosome. Mitochondria are also capable of generating ceramide via the action of reverse CDase [42, 101, 108].

### Sphingosine-1-phosphate and ceramide-1-phosphate

Phosphorylation/dephosphorylation reactions represent a mechanism through which cells respond to specific changes: the phosphorylated state of a molecule often exhibits effects that are diametrically different from those of the unphosphorylated state. Besides being used to resynthesize ceramide, sphingosine can be converted into sphingosine-1-phosphate (SP1) via sphingosine kinase, an enzyme that exists in the cytosol and ER [109, 110] (Fig. 2). The terminal catabolism of sphingosine involves the action of SP1 lyase, which degrades the SP1 to form ethanolamine phosphate and a fatty aldehyde [111]. Sphingosine is associated with growth arrest [112] whereas its phosphorylated form, SP1, is able to promote cell proliferation and prevent programmed cell death [110] (for a review [113]).

Ceramide and S1P that exert effects of opposite nature in their regulation of apoptosis, differentiation, proliferation and cell migration [114, 115]. The concentration of ceramide and S1P is counter-balanced by enzymes that convert one lipid to the other and their levels are believed to balance between cell viability and cell death.

However, this is not the only way the cell can balance to ensure tissue homeostasis. Ceramides can also be phosphorylated by the enzyme ceramide kinase (CERK) to form ceramide-1-phosphate (Cer1P) [116–119]. As expected, phosphorylation of ceramide in Cer1P allows a switch of ceramide properties: comprehensive studies indicate that Cer1P inhibits apoptosis and can induce cell survival [120–122].

CERK was first observed in brain synaptic vesicles [117] and found to be highly expressed in brain, heart, skeletal muscles and liver [116]. It appears that at least two different CERK isoforms exist in neural tissue, a calcium dependent enzyme at the plasma membrane level and a second cytosolic enzyme [123, 124]. The former enzyme localizes at synaptic-vesicles suggesting a possible role for CERK in neurotransmitter release [116, 117, 125].

CERK specifically utilizes ceramide transported to the Golgi apparatus by CERT [126]. Stable downregulation of CERT by RNA interference results in strong decrease in Cer1P levels, suggesting that Cer1P formation mostly relies on ceramide de novo synthesis [126]. Together with CERK and Cer1P phosphatases, CERT could modulate an appropriate balance between the intracellular levels of ceramide and Cer1P. However it is important to mention that short-term pharmacological inhibition of CERT appears to slow down SM synthesis without decreasing Cer1P synthesis [127], suggesting either an alternative route for delivery of ceramide to CERK at the Golgi complex or a process which is dependent on long-term responses.

Maintenance of equilibrium between ceramide and Cer1P seems to be crucial for cell and tissue homeostasis and accumulation of one or the other results in metabolic dysfunction and disease.

Recently, S1P was reported to function not only as an intracellular but also as an extracellular mediator of cell growth through endothelial-differentiation gene family receptors [128]. Cer1P could exert similar functions at the plasma membrane level. Further research is necessary to study if ceramide could reach the plasma membrane transported by CERT_L_ allowing plasmatic membrane CERK to form Cer1P.

## Plasma membrane, not just a lipid bilayer

### Structural organization of the membrane

The plasma membrane is the densest structure of eukaryotic cells and it defines the outer limit of the cell with its environment. Far from being a passive skin around a cell, plasma membranes are highly dynamic structures with a central role in a vast array of cellular processes [129, 130].

**Fig. 3:**
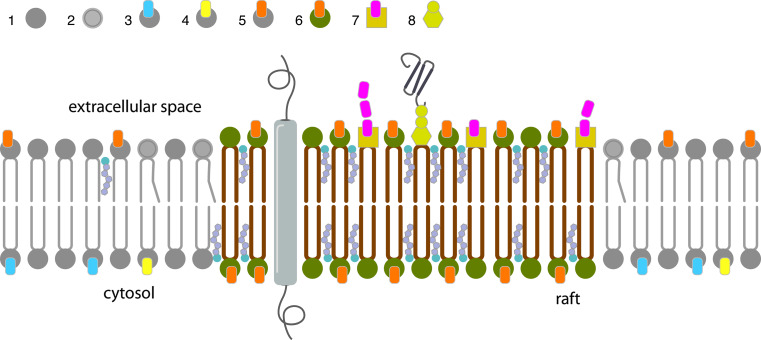
Schematic representation of lipid raft structures in a plasma membrane. The phospholipid bilayer of cellular plasma membranes contains many different lipid components such as glycerophospholipids, sphingolipids and cholesterol. The compositions of the inner and outer membrane leaflets are different. The cytoplasmic monolayer is largely composed of aminophospholipids as phosphatidylserine (*4*) and phosphatidylethanolamine (*3*). By contrast, the choline-containing lipids SM (*6*) and phosphatidylcholine (*5*) and a variety of glycolipids (*7*, *8*) are significant components of the exofacial leaflet of plasma membranes [45]. SM (*6*) together with cholesterol and different GSLs (*7*, *8*), form highly organized microdomains called lipid rafts on the plasma membrane. Since these microstructures are formed by lipid species with long saturated acyl chains, rafts are rigid platforms which float in the more fluid surrounding membrane that consists of phospholipids with saturated (*1*) and unsaturated (*2*) fatty acyl chains and less cholesterol. Lipids rafts are enriched in glycosylphosphatidylinositol (GPI)-anchored proteins (*8*) at their external surface and studded with transmembrane integral proteins

Plasma membrane of eukaryotic cells comprises three major classes of lipids: glycerophospholipids, sphingolipids and sterols, principally cholesterol [131]. Glycerophospholipids are the main building blocks of eukaryotic membranes and differ from sphingolipids (ceramide based lipids) in that they are built on a glycerol backbone [132]. Sphingolipid acyl chains are characteristically highly saturated, this allows them to pack tightly in the lipid bilayer and results in a liquid ordered phase with little opportunity for lateral movement or diffusion. This characteristic makes sphingolipids suitable to contribute heavily to the structure of the outer leaflet [30]. Conversely, glycerophospholipids are rich in unsaturated acyl chains that are typically kinked, this means they pack loosely thus increasing the fluidity of the lipid bilayer. The inner leaflet has a higher content of unsaturated phospholipids. This lipid asymmetry in membranes accounts for the greater fluidity of the inner layer relative to the outer layer (Fig. 3).

Sphingolipids molar ratio relative to glycerophospholipids and cholesterol varies within cell types. For instance, GSLs are a very minor component in certain cell types such as erythrocytes but they have been shown to be particularly abundant in neurons and oligodendrocytes where they make up 30 % of total lipids in myelin sheets [133, 134]. Cholesterol affects the consistency of the plasma membrane making the outer surface firm and decreasing its permeability [135]. With its rigid ring structure, cholesterol fills interstitial spaces between fatty acid chains of the nearest phospholipids, restricting their movement. At the same time cholesterol helps plasma membrane to maintain its fluidity, separating the long saturated fatty acid tails of phospholipids, avoiding their condensation. Despite the significance of ceramide metabolism in the synthesis and degradation of sphingolipids, ceramide content is normally very low in cell membrane and increases in ceramide concentration are highly localized and temporally regulated. The occurrence of ceramide in the lipid bilayer directly affects both the structural organization and the dynamic properties of the cell membrane [11, 136].

### Lipid raft

Many cellular processes such as endocytosis, exocytosis and membrane budding involve changes in membrane topology. While biological membranes are typically in a fluid or liquid-disordered state at physiological temperatures, combinatorial interactions between specific lipids drives the formation of dense, liquid-ordered domains, or ‘lipid rafts’ within membranes [13, 130, 137, 138] (Fig. 3). The characteristics of these microdomains differ from those of the whole membrane. They are generally enriched in lipids with saturated acyl chains, especially SM and cholesterol which pack tightly within the lipid bilayer [139, 140]. These separated regions seem to exist as preformed entities in the membrane of resting cells [141] and are present in different parts of the lipid bilayer [142].

The straight saturated acyl chains of sphingolipids in rafts are more extended than unsaturated chains of surrounding phospholipids and as a result lipid rafts extend 1 nm beyond the phospholipids background [143]. The isolation of biologically relevant lipid rafts is problematic. In the past, highly saturated lipid rafts have been isolated based on their detergent resistance [144]. More recently, it has been shown that these detergent resistant membranes (DRMs) are in fact a product of the extraction method and do not reflect any specific membrane structure. Therefore, it is important to recognize that rafts are not equivalent to DRMs [145]. The majority of studies have investigated lipid rafts mainly at the plasma membrane, due to their accessibility from the outside of the cell [146–148]. However many intracellular organelles contain raft-like domains [144, 149–152]. Membranes of the Golgi are rich in cholesterol/SM [153–155] and it has been suggested that rafts function in sorting of lipids and proteins in the secretory and endocytic pathways. In particular, raft like domains are thought to be abundant in the trans-Golgi [152, 156] and in late endosomes [151].

Lipid rafts are dynamic structures without any characteristic morphology [157]: during the steady state, rafts may be very small, nanometers in diameter [139, 158, 159] but upon proper stimuli they can coalesce into large domains making even micrometer-size rafts [159]. The fundamental principle by which lipid rafts exert their functions is a segregation or concentration of specific membrane proteins and lipids to form distinct microdomains [147] that represent specialized signaling organelles within the plasma membrane [160]. These dynamic membrane sites have been implicated in mechanisms of cell polarity [161], membrane trafficking including endocytosis [149, 162] and exocytosis [163–165] and in intracellular signaling [160, 166–168].

Proteins which localize into lipid rafts often show post-translational modifications with lipids such as glycosylphosphatidylinositol (GPI)-anchors, palmitoylation, prenylation, myristoylation, [169] or directly bind cholesterol or phospholipids as caveolins [138, 170] and annexins [171], respectively.

### Ceramide-enriched platforms

**Fig. 4:**
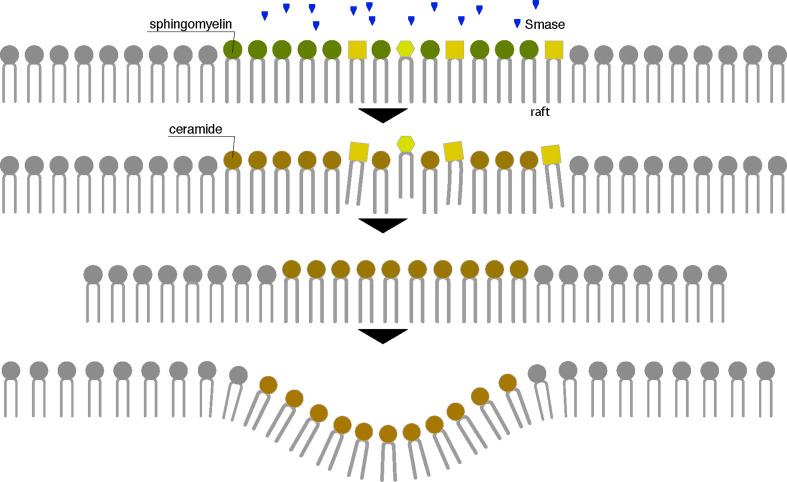
Scheme of lipid raft reorganization up in ceramide formation by SMase activity. Hydrolysis of SM through the enzyme Smase generates ceramide in the outer leaflet of the cell membrane. For its biochemical features, ceramide mixes poorly with the other rafts components and shows self-assembling capability in the membranous environment forming large distinct ceramide-enriched membrane platforms which serve to reorganize the cell membrane, resulting in clustering of activated receptor molecules

As a highly hydrophobic second messenger, ceramide presumably acts at the level of lipid rafts in transducing external signal. Rafts are the primary site of action of the enzyme SMase that releases ceramide from SM [172] (Fig. 4). The tight interaction between SM and cholesterol serves as the basis for raft formation. Ceramides, on the other hand, mix poorly with cholesterol and have a tendency to self associate and segregate into highly ordered microdomains [13, 173]. The nature of ceramide has a strong impact on membrane structure. In fact, long-chain saturated ceramide molecules, are intermolecularly stabilized by hydrogen bonding and van der Waal forces [25, 174] and form a liquid ordered domains that induce lateral phase separation of fluid phospholipid bilayers into regions of liquid-crystalline (fluid) phases. Moreover, the small size of ceramide polar headgroup results in a low hydration and allows ceramide molecules to pack tightly avoiding any interference with surrounding lipids [175]. In fact it has been shown that as little as 5 mol% ceramide is sufficient to induce ceramide partitioning in the lipid bilayer and to drive the fusion of small inactive rafts into one (or more) larger active ceramide-enriched membrane platforms [174].

Among lipids, DAG is structural similar to ceramide. DAG is produced in the cell membrane by hydrolysis of phosphatidylinositol 4,5-bisphosphate [176] and phosphatidylcholine [177]. Both are very minor components of membrane being formed and removed rapidly at specific locations in response to signaling. As well as ceramides, DAGs also give rise to phenomena of lateral phase separation in small domains within phospholipid bilayers. Both ceramide [178] and DAG [179] have a small polar head and a large hydrophobic region; they tend to bend the bilayer and to facilitate the formation of non-bilayer (non-lamellar) phases which are important for cellular processes such as pore formation, vesicle fusion and budding, as well as membrane protein function. Also, both lipids act as second messengers that play important roles in many signaling pathways. DAG is able to induce structural changes in membrane, more efficiently than ceramide, requiring as little as 2 mol% [180]. This difference in efficiency is likely due to the different physical properties of these lipids. It is though that the different proficiencies of ceramide and DAG for induction of membrane structural change through transient destabilization of the lamellar structures provide opportunity for fine control of membrane properties.

The ceramide-enriched membrane platforms serve as clustering components to achieve a critical density of receptors involved in signaling. In fact, rafts are too small to engage in membrane processes [160, 181]. This high density of receptors seems to be required for effective transmission of the signal into cells. For example, CD95 signaling is amplified a hundred-fold by the formation of ceramide-enriched membrane platforms [182].

The neuronal plasma membrane is particularly enriched in lipid rafts [183]. More than 1 % of total brain protein is recovered in a lipid raft fraction, whereas less than 0.1 % of total protein is associated with lipid raft isolated from non neuronal tissues [184]. In cultured neurons, lipid rafts are distributed throughout the cell surface including the soma and dendrites. As well as across cell types, lipid and protein raft composition differs according to neuronal developmental stage. Mature neuron lipid raft content is higher than that of immature neurons and astrocytes. [185]. Synaptic proteins such as synaptophysin or synaptotagmin localize in lipid rafts [186, 187] and lipid rafts are critical for maintaining the stability of synapses and dendritic spines [188]. Neurotransmitter signaling seems to occur through a clustering of receptors and receptor-activated signaling molecules within lipid rafts. Several lipid raft associated neurotransmitter receptors have been isolated from brain tissues, examples include: nicotinic acetylcholine receptors [189], gamma aminobutyric acid type B receptors [190], α-amino-3-hydroxy-5-methyl-4-isoxazolepropionic acid receptor and *N*-methyl-d-aspartate receptors [188, 191, 192]. Aberrant organization of SM and cholesterol in rafts has been linked to loss of synapses and changes in nerve conduction [188]. Depletion of sphingolipids or cholesterol leads to gradual loss of inhibitory and excitatory synapses and dendritic spines [188]. Rafts also play an important role in neuronal cell adhesion [193], localization of neuronal ion channels [194, 195] and axon guidance [196]. In oligodendrocytes, rafts mediate the interaction between myelin associated glycoprotein on myelin and its receptor on neurons [197].

## Ceramide signaling in apoptosis

Apoptosis is an essential process for normal embryonic development and to maintain cellular homeostasis within mature tissues. A proper balance between regulation of normal cell growth and cell death is the basis of life. Deregulated apoptosis is a feature of most pathological conditions such as neurodegeneration, auto immune disorders and cancer. In neurodegenerative diseases such as Alzheimer’s, Parkinson’s, Huntington’s and Prion’s diseases aggregated misfolded proteins contribute to the neuronal pathogenesis; in multiple sclerosis, autoimmune mechanisms accompany the demyelination; in HIV-associated dementia, viral products are crucial for neuronal demise. Factors affecting neurodegeneration can differ, but these devastating disorders are all characterized by a massive loss of specific populations of neurons or damage to neuronal transmission.

Premature death of terminally differentiated cells such as neurons and oligodendrocytes results in progressive and irreversible functional deficits since these post mitotic cells cannot be easily replaced [198]. The role of ceramide in apoptosis is extensive and complex and despite intense investigations remains controversial [199]. An increase of ceramide levels leads to cell death [200, 201]; in contrast, depletion of ceramide can reduce the progression of apoptosis [202–204]. However, ceramide is indispensable for proper function of the central nervous system (CNS) [205–207]. Ceramide levels inside the cell determine its dual role: protection and cell sustenance at low concentrations but death and threat when over produced. This outlines the importance for cells to maintain a strict ceramide balance by a tight regulation of sphingolipid based signaling networks.

**Fig. 5:**
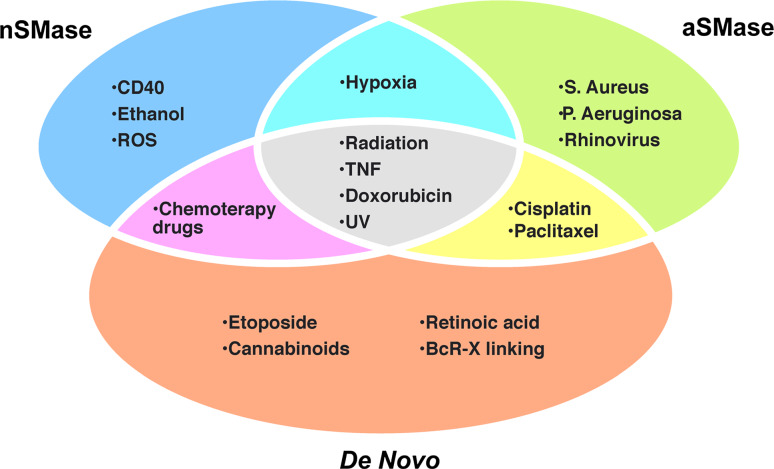
Ceramide production occurs in response to diverse apoptotic stimuli and with different mechanisms. Many inducers of cell death activate one or more ceramide generation pathways. For example both SM hydrolysis (by either a nSMase or an aSMase) and the de novo pathway have been implicated in the action TNFα, radiation, doxorubicin and UV. Ultimately, ceramide production results in cell death regardless of the pathway

Ceramide can induce apoptosis via different routes and different intracellular organelles are the target of its action. SM hydrolysis by neutral and/or acid SMases is known to be a very important pathway for production of pro-apoptotic ceramides [208]. However, the de novo synthesis pathway has also been reported to be relevant in the generation of a signaling pool of ceramide leading to cellular apoptosis [209–211]. These two pathways can induce apoptosis independently or jointly (Fig. 5).

SM hydrolysis generates a rapid and transient increase of ceramide and results in formation of ceramide-enriched membrane platforms. In contrast, the ceramide de novo pathway requires multiple enzymatic steps and it is responsible for a slow but robust accumulation of ceramide over a period of several hours.

SMase activation occurs in response to stimulation of cell surface receptors of the tumor necrosis factor (TNF) upon the binding with specific ligands such as TNF alpha, TNF-related apoptosis-inducing ligand (TRAIL) and Fas ligands.

SM hydrolysis in response to TNF signals involves both nSMase and aSMase but their activation occurs through different mechanisms [212, 213]. The cytoplasmic tail of the TNFR1 contains two distinct portions that differently associate with nSMase or aSMase [214, 215]. Activation of aSMase requires the C-terminal of the TNFR1 identified as death domain (DD) [216]. DD associates with the adaptor protein TRADD (TNF receptor 1-associated death domain) that together with another cytoplasmic protein, termed FADD/MORT-1 [217] induces activation of aSMase [218]. ASMase is normally present in the endosomal/lysosomal compartment. However, upon phosphorylation by protein kinase C, aSMase translocates from its intracellular locations to the plasma membrane where it reaches SM [219]. ASMase is reported to be functional at physiological pH after translocation to the plasma membrane [220]. The ceramide produced by aSMase activates the aspartyl protease cathepsin D [221] that can subsequently cleave the pro-apoptotic Bcl-2 family member Bid. Activation of Bid induces cytochrome c release from mitochondria [222] and activation of caspase-9 and -3, leading to apoptotic cell death by the intrinsic pathway [223].

Conversely, activation of nSMase requires a short motif adjacent to the DD of TNFR1, called neutral sphingomyelinase domain (NSD). The NSD binds an adaptor protein, FAN (factor associated with nSMase) which couples nSMase to TNFR1 [224]. The ceramide generated by nSMase leads to the activation of ceramide-activated protein kinase (CAPK) [14] and ceramide-activated protein phosphatases (CAPPs) [225], direct downstream targets of ceramide. CAPK, Ser/Thr protein kinase, is involved in the mitogen-activated protein kinase (MAPK) cascades that induce the extracellular-signal regulated kinases (ERK) activation. ERK cascade leads to cell cycle arrest and cell death.

CAPPs, which comprise the serine threonine protein phosphatases PP1 and PP2A [226], mediate the effect of ceramide through dephosphorylation and inactivation of several substrates, such as retinoblastoma gene product (RB) [227], Bcl-2 and Akt [228] and through downregulation of the transcription factors c-Myc [229] and c-Jun [230].

Although aSMase and nSMase seem to induce death receptor dependent and independent mediated apoptosis through apparently separate mechanisms, both enzymes are activated by the same stimuli, i.e. UV light [231], hypoxia [232, 233], radiation [204, 234], TNF-related apoptosis-inducing ligands [235] and the DNA-damaging drug doxorubicin [236]. Disruption of rafts or prevention of ceramide generation by inactivation of aSMase, renders cells resistant to receptor clustering and apoptosis indicating that aSMase plays an important role in death receptor-mediated apoptosis [2, 237, 238]. Accordingly, aSMase-deficient mice are resistant to the induction of apoptosis by CD95 [239] and TNF alpha signaling [240].

Selective activation of nSMase has been reported to occur for some apoptotic stimuli as CD40 [241], ethanol [242], free oxygen radicals [243] and chemotherapy drugs [244] (Fig. 5). In contrast, specific activation of aSMase with subsequent formation of ceramide-enriched membrane domains occurs after infection with *Pseudomonas aeruginosa* [245], *Staphylococcus aureus* [246] or rhinovirus [247].

Instead, exposition to the chemotherapeutic agent etoposide [211] and cannabinoids [248], retinoic acid [249] and B cell receptor (BcR)-induced apoptosis [250] all involve a large increase in ceramide levels formed specifically through the de novo pathway. However, the downstream targets of the de novo ceramide dependent cell death are largely unknown.

In conclusion, evidence suggests that ceramide acts either by changing the physical state and organization of cellular membranes or by direct binding and activation of target proteins. The spatial reorganization of plasma membrane driven by generation of ceramide may serve to cluster signaling molecules and to amplify death signaling. However, rather than a specific mechanism for apoptosis induction, this process appears to represent a generic mechanism for transmembrane signaling. In fact, receptors that are not involved in apoptosis (IL5, LFA 1, CD28, CD20) [251] can activate the SMase signaling pathway with subsequent raft clustering into microdomains. Beside its effect at the level of cellular membranes, ceramide is capable of direct binding with components that lead to death as CAPP, CAPK, protein kinase C-ξ, cathepsin D [252] and mediate induction of signaling cascades that lead to apoptosis, growth arrest and inflammation.

## Aging

Sphingolipids hold a major role in regulating development and lifespan [253] and deregulation in sphingolipid metabolism increase the risk and progression of age-related neurodegenerative disease [254, 255]. Since ceramide is the core of sphingolipids, its contribution to cellular pathophysiology is object of intense study. A close connection between ceramide levels and aging comes from studies carried on *Saccharomyces cerevisiae* where a gene involved in ceramide synthesis has been identified as a regulator of yeast longevity. This gene called longevity assurance homolog 1 (LAG1), together with LAC1, functions as a key components of CerS in vivo and in vitro [256] and its lost correlates with a marked increase in yeast lifespan [257]. The human homolog LAG1Hs (CerS1) is highly expressed in the brain, testis and skeletal muscles and specifically generates C18-ceramide [46]. This conclusion seems to be supported by cell culture studies where overexpression of CerS1 with increased C18-ceramide generation resulted in apoptosis [258]. Interestingly, C18-ceramide generated by CerS1 was found to downregulate the expression of the enzyme telomerase [259]. Telomerase functions by elongating the end of existing chromosomes and thus preventing cellular senescence. Since cellular aging is dependent on cell division, these enzymes play a critical role in long-term viability of highly proliferative organ systems [260]. Specifically C18-ceramide is able to mediate a negative regulation of the human telomerase reverse transcriptase (hTERT) promoter, whereas different ceramides generated by other ceramide synthases do not have such a function. Telomerase is expressed in neurons in the brains of rodents during embryonic and early postnatal development and is subsequently downregulated [261]. Terminally differentiated neurons are postmitotic, therefore there is not need to maintain the telomere length [262]. However, telomerase is constitutively expressed in restricted regions of the hippocampus and the olfactory bulbs which are continuously supplied with neural stem and progenitor cells [263]. These cells are required for adult neurogenesis throughout life because they produce new neurons and support brain cells. Therefore, besides the telomeric roles, telomerase was found to protect the post-mitotic neuronal cells from stress-induced apoptosis and may serve a neuron survival-promoting function in the developing brain and be important for regulating normal brain functions. Thus, the regulation that C18-ceramide seems to exert on telomerase expression may contribute to increase neuronal vulnerability of the adult brain in various age-related neurodegenerative disorders.

Several studies support the role of ceramide in inducing senescence and in activating genetic/biochemical pathways involved with aging. Accumulation of ceramide occurs normally during development and aging in single cells [264] and young cells treated with exogenous ceramide exhibit a senescent-like phenotype [265].

In addition, a significant change in ceramide metabolic enzyme activities seems to occur in specific organs or even in specific cell types with aging [264, 266]. The activities of the sphingolipid catabolic enzymes (SMase and CDase) seem to change more robustly than that of the anabolic enzymes (SMS and CerS).

ASMase and nSMase activity significantly increase in rat brain during aging [267] demonstrating that aging is accompanied by an increase in SM turnover. NSMase was also reported to be dramatically activated in senescent fibroblasts [264]. ACDase, nCDase and alCDase activities are increased specifically in brain tissue from aging rats and among the isoforms of CDases, alCDase shows the highest activity [267]. Increase in the CDase activity in kidney and brain indicates an increase in the production of sphingosine and its contribution toward aging in these tissues. In contrast, CerS shows a lower activity, suggesting a minor contribution of ceramide de novo synthesis to ceramide accumulation [267].

## Ceramide and neurodegeneration

### Lipid storage disorders

Ceramide is defined as a central element in the metabolic pathways of sphingolipids. All sphingolipids are synthesized from ceramides and are hydrolyzed to ceramides. In addition to CDase and SMase, there are other hydrolytic enzymes which hydrolyze complex sphingolipids producing ceramides as product. More than ten specific acid exohydrolases are responsible for intracellular GSLs digestion in a stepwise action that takes place within the lysosome. Deficiency or malfunctioning of one of these enzymes results in accumulation of the corresponding lipid substrate in the lysosomal compartment leading to cellular enlargement, dysfunction and death. Due to its high synthesis of lipids, the brain is the organ mainly affected by accumulation of lipid products. Their abnormal storage and slow turnover results in severe dementia and mental retardation. Inherited metabolic disorders which have been linked to lysosomal dysfunction belong to a family of diseases identified as lysosomal storage disorders (LSDs).

LSDs include Farber’s disease, caused by the dysfunction of aCDase; Krabbe’s disease (Globoid leukodystrophy), caused by the absence of galactosylceramidase (GalCer/3-galactosidase); Gaucher’s disease due to the absence of glucosylceramidase (GlcCer/3-glucosidase) and Niemann–Pick disease (NP) characterized by the absence of aSMase.

### Farber’s disease

Farber’s disease is an inherited disorder characterized by high levels of ceramides due to deficient activity of lysosomal aCDase [268]. The rate of ceramide synthesis is normal but ceramide resulting from degradation of complex sphingolipids cannot be hydrolyzed and it is confined into the lysosomal compartment [269]. There is a significant correlation between the ceramide accumulated in situ and the severity of Farber disease [270]. The abnormal ceramide storage in the brain results in neuronal dysfunction, leading to progressive neurologic deterioration. The inflammatory component of this disease consists in chronic granulomatous formations [271]. Granuloma are small areas characterized by the presence of lymphocytes, monocites and plasma cells [272] and appear to result from a dysregulation of leukocyte functions. However, the sequence of molecular mechanisms leading from defect in ceramide metabolism to leukocyte dysregulation is still unknown.

### Krabbe’s disease and Gaucher’s disease

Krabbe’s disease is a disorder involving the white matter of the central and peripheral nervous systems. It is characterized by a deficiency in the lysosomal enzyme galactosylceramidase which removes galactose from galacto-ceramide derivatives. Galactosylceramidase is necessary to digest galactosylceramide, a major lipid in myelin forming oligodendrocytes and Schwann cells [273]. Abnormal storage of galactosylceramide due to the lack of this enzyme leads apoptosis of myelin forming cells with a complete arrest of the myelin formation and consequent axonal degeneration. This accounts for the severe degeneration of motor skills observed in the disease. Another GSL called psychosine (the deacylated form of galactosylceramide, also known as galactosylsphingosine) is normally broken down by galactosylceramidase. Psychosine is present in the normal brain tissues at very low concentrations, owing to its rapid breakdown to sphingosine and galactose by galactosylceramidase. In its absence, psychosine accumulates in the brain acting as cytotoxic metabolite [274] and therefore contributing to oligodendroglial cell death. Psychosine was also found to cause axonal degeneration in both the central and peripheral nervous system by disrupting lipid rafts [275]. Myelin and/or oligodendrocyte debris produced by oligodendrocyte death in Krabbe’s disease activates microglial cells, resident macrophages in the brain, which are the primary mediators of neuroinflammation [276]. Because a pathological hallmark of this rapidly progressive demyelinating disease is the presence of multinucleated macrophages (globoid cells) in the nervous system [277] the disease is also known as globoid cell leukodystrophy. However, the function of these cells is unclear.

Gaucher’s disease is characterized by the lysosomal accumulation of GlcCer due to defects in the gene encoding the lysosomal hydrolase glucosylceramidase [278]. In the brain, GlcCer accumulates due to the turnover of complex lipids during brain development [279]. The cells most severely affected are neurons because they process large amounts of gangliosides which are components of their membranes and synapses. The demyelination or disrupt of the membrane structure may be the major consequence of these diseases and it is conceivable that a change in the ceramide at the plasma membrane level may contribute to these disorders. Enzymes involved in ganglioside degradation are highly expressed in brain tissue and are of particular importance in the first few years of life when axons elongate, dendrites branch and synapses develop [279]. Deficiency of these enzymes causes neuronal storage of gangliosides leading to loss of neurons and their axons, resulting in cortical atrophy and white matter degeneration. Cells and organs that do not process large amounts of gangliosides are either normal or show mild storage without cell damage.

### Niemann Pick’s disease

Defects in SM degradation results in a neurodegenerative condition known as NP. This kind of disorder exists in three major forms. Both NP type A and type B are caused by defects in lysosomal aSMase activity. Affected individuals cannot convert SM to ceramide [280] and alteration of the ceramide–SM ratio, rather than SM accumulation, is likely responsible for the onset of the disease. The importance of SM as a source of ceramide is indicated by the fact that activation of the aSMAase occurs in response to numerous signals within the cell and the production of ceramide is critical for an appropriate signaling cascade. NP type C diseases are caused by defects in a protein, NP C1 protein, which is located in membranes inside the cell and is involved in the movement of cholesterol and lipids within cells [281]. A deficiency of this protein leads to the abnormal accumulation of cholesterol and glycolipids in lysosomes and leads to relative deficiency of this molecule for steroid hormones synthesis.

### Neurodegenerative dementia: Alzheimer’, Parkinson’ and Prion’s diseases

Neural cells are very complex morphologically. The large plasma membrane surfaces of neurons are important for neuronal trafficking, neuron–neuron communication and signaling transduction. During aging and neurodegeneration membrane dysregulation and dysfunction are often found. These alterations in membrane microenvironment occur very early in the CNS [282, 283]. Heightened oxidative stress has a profound impact upon membrane lipid-protein organization and signal transduction [284]. These changes might be at the basis of diseases such as Alzheimer’s disease, Parkinson’s disease (PD), synucleinopathies, prion diseases, and other dementias.

Lipid rafts have been shown to be involved in the regulation of APP processing and in Aβ peptide formation [285], and represent the principal sites within the membrane where β-secretase and γ-secretase generate the pathological amyloid β peptide [286–290].

Other lipid raft components, such as the gangliosides GM1 and GM2, have been associated with induction of Aβ transition from a α-helix-rich structure to a β-sheet-rich conformation [291, 292]. Ganglioside binding with Aβ accelerates Aβ fibril formation [293] which gradually causes membrane raft disruptions and thereby has profound consequences on signal transduction and neurotransmission.

Prion protein (PrPc) is a GPI-anchored protein [294] and together with its pathological variant associates with lipid rafts [295]. Moreover, the conversion of PrPc into PrPSc has been shown to occur in these membrane domains [296]. Alpha-synuclein associates specifically with lipid rafts [297] and abnormalities of lipid rafts in the frontal cortex occur during the development of PD pathology [298]. Massive modification of fatty acid content gives rise to more viscous and liquid ordered rafts in PD brains than in the age-matched control group [298]. Also, lipid rafts from AD brains exhibit aberrant lipid profiles compared to healthy brains [299].

Similar lipid changes are also observed in epilepsy and ischemia/stroke [300, 301]. Elevations of intracellular ceramide levels, which may in turn be associated with induction of apoptotic cell death, have been reported in brain tissues and CSF of AD brain [302] together with reduced SM [303] and altered ganglioside levels [304]. In line with this, an increase of aCDase [305] and aSMase activity [306] has been detected in the brain of AD patients.

The key enzyme in ceramide de novo synthesis, SPT, is regulated by APP processing [307] suggesting that this could be one of probably many mechanisms responsible for the alterations in lipid metabolism at the plasma membrane.

## Conclusions

Ceramide is an important signaling molecule involved in the regulation of cell development, growth and apoptosis. In healthy cells ceramide metabolism is finely tuned and precisely coordinated and the level of ceramide generated can dictate whether development is stimulated or whether apoptosis is induced. Ceramide is beneficial for early growth and development of neuronal cells [308, 309] and at low levels it has trophic effects promoting cell survival and division. Initial abnormal formation of ceramide can potently induce more ceramide accumulation in a self-sustaining way [200, 310] that results to be toxic and supports pro-apoptotic actions in many cell types [311]. This induces drastic consequences leading to tissue damage and organ failure [312]. The mechanisms by which ceramide induces these disparate effects is not known, but may involve its effects in membrane structure and/or activation of different downstream signaling pathways.

These apparently contradictory roles can be understood only when we consider ceramide formation as a balanced and vulnerable system. This is, however, a fine line to tread and deviation in either direction can have drastic consequences. Where ceramide is concerned, growth arrest or apoptosis are only a slight tilt away.
